# Maternal Acetate Supplementation Reverses Blood Pressure Increase in Male Offspring Induced by Exposure to Minocycline during Pregnancy and Lactation

**DOI:** 10.3390/ijms23147924

**Published:** 2022-07-18

**Authors:** Chien-Ning Hsu, Hong-Ren Yu, Julie Y. H. Chan, Wei-Chia Lee, Kay L. H. Wu, Chih-Yao Hou, Guo-Ping Chang-Chien, Sufan Lin, You-Lin Tain

**Affiliations:** 1 Department of Pharmacy, Kaohsiung Chang Gung Memorial Hospital, Kaohsiung 833, Taiwan; cnhsu@cgmh.org.tw; 2 School of Pharmacy, Kaohsiung Medical University, Kaohsiung 807, Taiwan; 3 Department of Pediatrics, Kaohsiung Chang Gung Memorial Hospital and Chang Gung University College of Medicine, Kaohsiung 833, Taiwan; yuu2002@cgmh.org.tw; 4 Institute for Translational Research in Biomedicine, Kaohsiung Chang Gung Memorial Hospital, Kaohsiung 833, Taiwan; jchan@cgmh.org.tw (J.Y.H.C.); klhwu@cgmh.org.tw (K.L.H.W.); 5 Department of Urology, Kaohsiung Chang Gung Memorial Hospital, Kaohsiung 833, Taiwan; dinor666@ms32.hinet.net; 6 Department of Seafood Science, National Kaohsiung University of Science and Technology, Kaohsiung 811, Taiwan; chihyaohou@webmail.nkmu.edu.tw; 7 Center for Environmental Toxin and Emerging-Contaminant Research, Cheng Shiu University, Kaohsiung 833, Taiwan; guoping@csu.edu.tw (G.-P.C.-C.); linsufan2003@csu.edu.tw (S.L.); 8 Super Micro Mass Research and Technology Center, Cheng Shiu University, Kaohsiung 833, Taiwan; 9 Institute of Environmental Toxin and Emerging-Contaminant, Cheng Shiu University, Kaohsiung 833, Taiwan

**Keywords:** developmental origins of health and disease (DOHaD), gut microbiota, short chain fatty acid, acetate, minocycline, hypertension, inflammation, nitric oxide

## Abstract

Emerging evidence supports that hypertension can be programmed or reprogrammed by maternal nutrition. Maternal exposures during pregnancy, such as maternal nutrition or antibiotic use, could alter the offspring’s gut microbiota. Short-chain fatty acids (SCFAs) are the major gut microbiota-derived metabolites. Acetate, the most dominant SCFA, has shown its antihypertensive effect. Limited information exists regarding whether maternal acetate supplementation can prevent maternal minocycline-induced hypertension in adult offspring. We exposed pregnant Sprague Dawley rats to normal diet (ND), minocycline (MI, 50 mg/kg/day), magnesium acetate (AC, 200 mmol/L in drinking water), and MI + AC from gestation to lactation period. At 12 weeks of age, four groups (*n* = 8/group) of male progeny were sacrificed. Maternal acetate supplementation protected adult offspring against minocycline-induced hypertension. Minocycline administration reduced plasma acetic acid level, which maternal acetate supplementation prevented. Additionally, acetate supplementation increased the protein level of SCFA receptor G protein-coupled receptor 41 in the offspring kidneys. Further, minocycline administration and acetate supplementation significantly altered gut microbiota composition. Maternal acetate supplementation protected minocycline-induced hypertension accompanying by the increases in genera *Roseburia*, *Bifidobacterium*, and *Coprococcus*. In sum, our results cast new light on targeting gut microbial metabolites as early interventions to prevent the development of hypertension, which could help alleviate the global burden of hypertension.

## 1. Introduction

During pregnancy, maternal nutrition plays a decisive role in fetal growth and development. A maternal nutritional imbalance has been known to permanently change the structure and function of specific organs in the offspring, leading to many adult diseases [1]. This concept is referred to as the developmental origins of health and disease (DOHaD) [2]. Conversely, nutritional interventions targeting early life programming can reverse adverse programming processes, namely reprogramming, to avert adult diseases of developmental origins [3,4].

Increasing evidence supports that hypertension can be programmed or reprogrammed by maternal nutrition [1,3]. Maternal exposures during pregnancy, such as maternal nutrition or medication use, could impact microbial transmission resulting in long-term consequences to how the offspring’s gut microbiome develops in later life [5]. Metabolites derived from gut microbiota are suggested to play a role in the interplay between microbial dysbiosis and blood pressure (BP) [6,7,8]. Short-chain fatty acids (SCFAs) are volatile fatty acids produced by the bacterial fermentation of carbohydrates [9]. During pregnancy, SCFAs act as signaling molecules in relation to maternal metabolism through G-protein receptors [10]. Circulating SCFAs can also regulate their receptors located in the kidneys to regulate BP [11]. Additionally, alterations of gut microbiota are related to T helper 17 (Th17)-mediated immunity and renal inflammation in hypertension [12,13].

In addition to maternal malnutrition, the use of certain medications such as antibiotics during gestation increase the risk of getting high BP later in life [14]. Despite antibiotics being life-saving medicines for pregnant women to control bacterial infections, the use of antibiotics may perturb the gut microbiota of mothers and their offspring [15]. Both positive and negative consequences of gut microbiota altered by antibiotics have been reported in the context of hypertension, according to various types of antibiotics and different genotypes [16,17]. Minocycline, a tetracycline, has been reported to reduce BP accompanied by the restoration of dysbiotic gut microbiota in spontaneously hypertensive rats (SHR) [17]. However, we previously found that maternal minocycline administration caused a rise in BP in adult male rat progeny accompanied by a reduction in plasma SCFA levels [18].

Interventions targeting early life programming opens a door for the treatment and prevention of hypertension. SCFAs, the most commonly produced postbiotics [19], have been used as a reprogramming strategy for prevention of developmental programming of hypertension [20]. The most abundant SCFA is acetate, which can induce vasodilation and lower BP [9]. Prior research indicates that acetate supplementation prevents the development of hypertension by either the restoration of dysbiotic gut microbiota, reduction in inflammation, or regulation of genes involving in BP control [21,22,23]. However, the protective effects of acetate against maternal minocycline-induced hypertension remain unknown. The objective of this study is to evaluate whether acetate supplementation can protect maternal minocycline-induced hypertension in adult offspring through shifts in the gut microbiota compositions, regulation of SCFAs and their receptors, and reduction in renal inflammation.

## 2. Results

### 2.1. Body Weight and Blood Pressure

**Figure 1:**
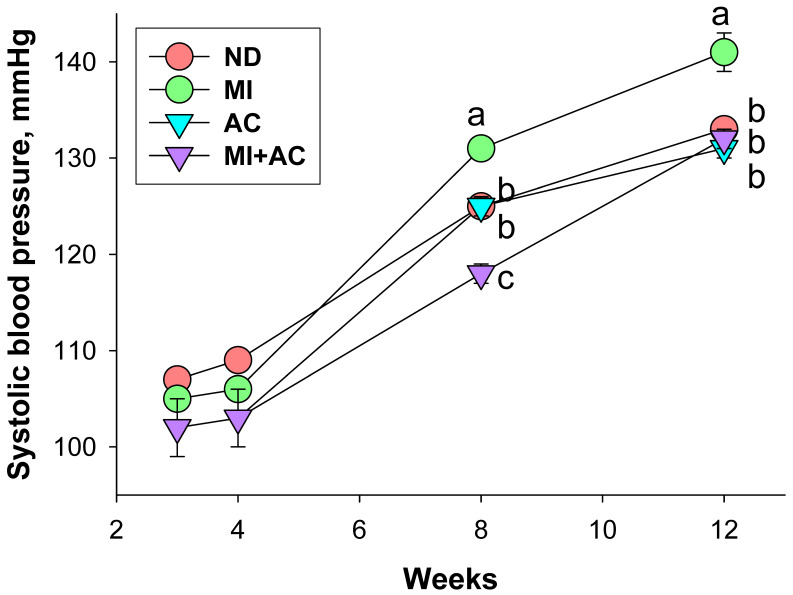
Effect of minocycline administration (MI) and acetate supplementation (AC) on systolic blood pressure in offspring from 3 to 12 weeks of age (*n* = 7–8/group). The letters a, b, and c denote significant differences between groups (*p* < 0.05, two-way ANOVA). ND = rats received vehicle (control); MI = rats received minocycline; AC = rats received acetate; MI + AC = rats received minocycline and acetate administration.

**Figure 2:**
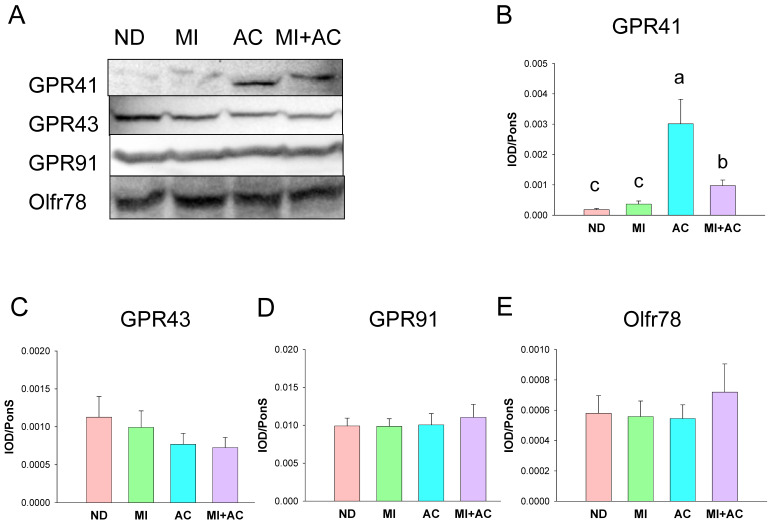
Representative Western blots and relative abundance of (**A**) short-chain fatty acid (SCFA) receptor, (**B**) G protein-coupled receptor 41 (GPR41), (**C**) GPR43, (**D**) GPR91, and (**E**) olfactory receptor 78 (Oflr78) in 12-week-old offspring kidneys (*n* = 7–8/group). The letters a, b, and c above the bars denote significant differences between groups (*p* < 0.05, one-way ANOVA). ND = rats received vehicle (control); MI = rats received minocycline; AC = rats received acetate; MI + AC = rats received minocycline and acetate administration.

We divided pregnant rats into four groups and treated them as follows during pregnancy and lactation periods: (1) ND, vehicle; (2) MI, minocycline; (3) AC, acetate, and (4) MI + AC, minocycline plus acetate administration. A pup was seen dead at 1 week after birth in the AC group (Table 1). The body weight (BW) of AC and MI + AC groups was lower compared to the ND and MI groups. The offspring in the MI + AC group have the highest kidney weight (KW)-to-BW ratio among all groups. The BP of rat progeny showed that minocycline administration caused an elevation in systolic BP (SBP) determined between weeks 8 and 12, which was prevented by acetate supplementation (Figure 1). We observed that SBP and the mean arterial pressure were highest in the MI group among all groups at 12 weeks of age. Taken together, results from Table 2 and Figure 2 revealed that maternal minocycline administration caused hypertension in adult progeny, which was prevented by acetate supplementation during gestation and lactation. Additionally, minocycline administration induced an increase in plasma creatinine level, an index of renal function, in the MI group, which was restored by acetate supplementation.

### 2.2. SCFA Levels and SCFA Receptors

We first determined whether minocycline administration and acetate supplementation affected circulating SCFA levels and their receptors in the kidneys. Table 2 illustrates that maternal minocycline administration causes a reduction of plasma acetic acid level, which was prevented by acetate supplementation. Additionally, both MI and AC had a negligible effect on plasma levels of propionic acid, isobutyric acid, butyric acid, isovaleric acid, and valeric acid.

We analyzed the expressions of four SCFA receptors in the offspring kidney, including G protein-coupled receptor 41 (GPR41), GPR43, GPR91, and olfactory receptor 78 (Oflr78). Figure 2 shows acetate supplementation remarkably increased the renal protein level of GPR41 in the AC and MI + AC groups. However, minocycline administration or acetate supplementation had little influence on GPR43, GPR91, and Oflr78 protein abundance in the kidneys.

### 2.3. Cytokine Concentrations in the Kidneys

**Figure 3:**
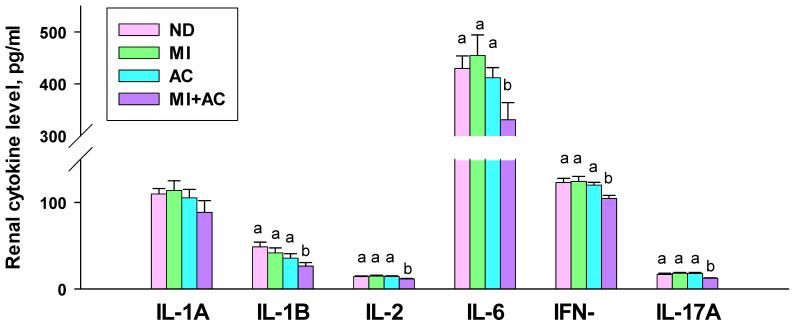
Effect of minocycline administration (MI) and acetate supplementation (AC) on cytokine levels in offspring at 12 weeks of age (*n* = 7–8/group). The letters a and b above the bars denote significant differences between groups (*p* < 0.05, one-way ANOVA). ND = rats received vehicle (control); MI = rats received minocycline; AC = rats received acetate; MI + AC = rats received minocycline and acetate administration.

We measured cytokines interleukin (IL)-1A, IL-1B, IL-2, IL-6, IL-17A, and interferon-γ (IFN-γ) in the offspring kidneys. As shown in Figure 3, the renal level of IL-1A was not different among the four groups. IL-1B and IL-6 levels were lower in the MI + AC group compared to the ND and MI groups. Additionally, renal IL-2, IFN-γ, and IL-17A levels were lowest in the MI + AC group compared to the other three groups.

### 2.4. Gut Microbiota Composition

**Figure 4:**
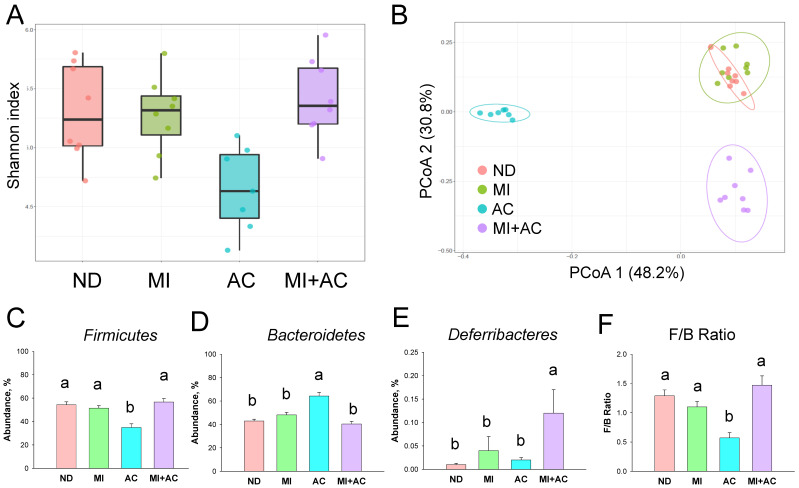
Effect of minocycline administration (MI) and acetate supplementation (AC) on the gut microbiota in 12-week-old male progeny: (**A**) α-diversity using the Shannon index; (**B**) β-diversity using principal coordinate analysis (PCoA); phylum level relative abundance of (**C**) Firmicutes, (**D**) Bacteroidetes, and (**E**) Deferribacteres; (**F**) the ratio of Firmicutes to Bacteroidetes (F/B) (*n* = 7–8/group). The letters a and b above the bars denote significant differences between groups (*p* < 0.05, one-way ANOVA). ND = rats received vehicle (control); MI = rats received minocycline; AC = rats received acetate; MI + AC = rats received minocycline and acetate administration.

We then explored α- and β-diversity metrics to evaluate the influence of minocycline administration and acetate supplementation on the total gut microbial community. Microbial α-diversity (Shannon index) was not significantly different between groups (Figure 4A). We further implemented the β-diversity metric, the Principal Coordinate Analysis (PCoA), to compare the microbial community similarity. We used the unweighted UniFrac metric to determine the distance between samples and PCoA to visualize the data. This is the percentage variance explained by each axis: PCoA 1 = 48.2% and PCoA 2 = 30.8%. The scatterplots of the PCoA analysis are depicted in Figure 4B and reveal significant clustering according to the study group, except the ND and MI groups are not completely separated. These data indicate that acetate supplementation, both individually and together with MI, significantly changed gut microbiota composition. The phylum level of *Firmicutes* was lower in the AC group compared to the ND and MI groups (Figure 4C), while this reduction was restored in the MI + AC group. Conversely, the phylum level of *Bacteroidetes* was highest in the AC group compared to the other three groups (Figure 4D). Additionally, the MI + AC group had the highest phylum level of *Deferribacteres*. The ratio of *Firmicutes*/*Bacteroidetes* (F/B), a microbial marker related to hypertension [1], was significantly reduced by acetate supplementation (Figure 4E).

**Figure 5:**
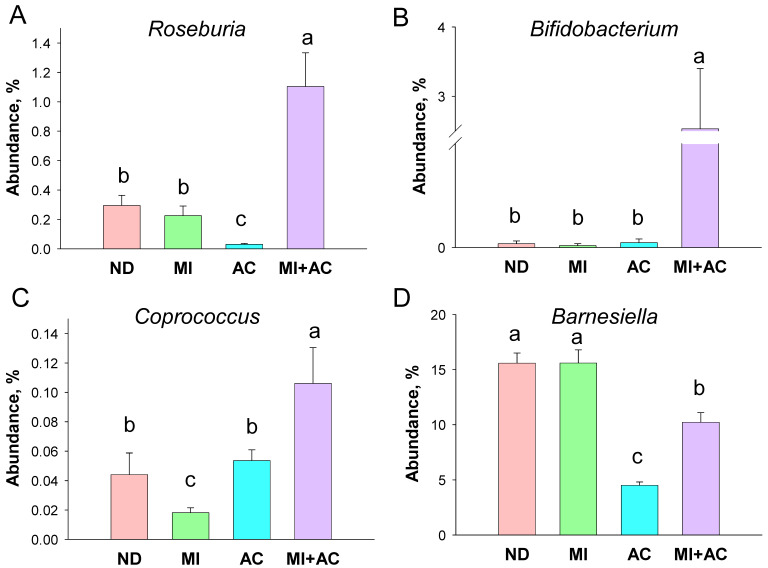
Effect of minocycline administration (MI) and acetate supplementation (AC) on the gut microbiota in 12-week-old male progeny: genus level relative abundance of (**A**) *Roseburia*, (**B**) *Bifidobacterium*, (**C**) *Coprococcus*, and (**D**) *Barnesiella* (*n* = 7–8/group). The letters a, b, and c above the bars denote significant differences between groups (*p* < 0.05, one-way ANOVA). ND = rats received vehicle (control); MI = rats received minocycline; AC = rats received acetate; MI + AC = rats re-ceived minocycline and acetate administration.

At the genus level, the abundance of *Roseburia* was affected by acetate supplementation (Figure 5A). The AC group had the lowest while the AC + MI group displayed the highest genus level of *Roseburia* among the four groups. Additionally, the relative abundance of genus *Bifidobacterium* was significantly augmented by MI + AC intervention (Figure 5B). As a result, acetate supplementation increased the abundance of *Coprococcus* in the AC and MI + AC group vs. MI group (Figure 5C). Moreover, acetate supplementation caused a reduction in the genus level of *Barnesiella* in the AC and MI + AC groups (Figure 5D).

**Figure 6:**
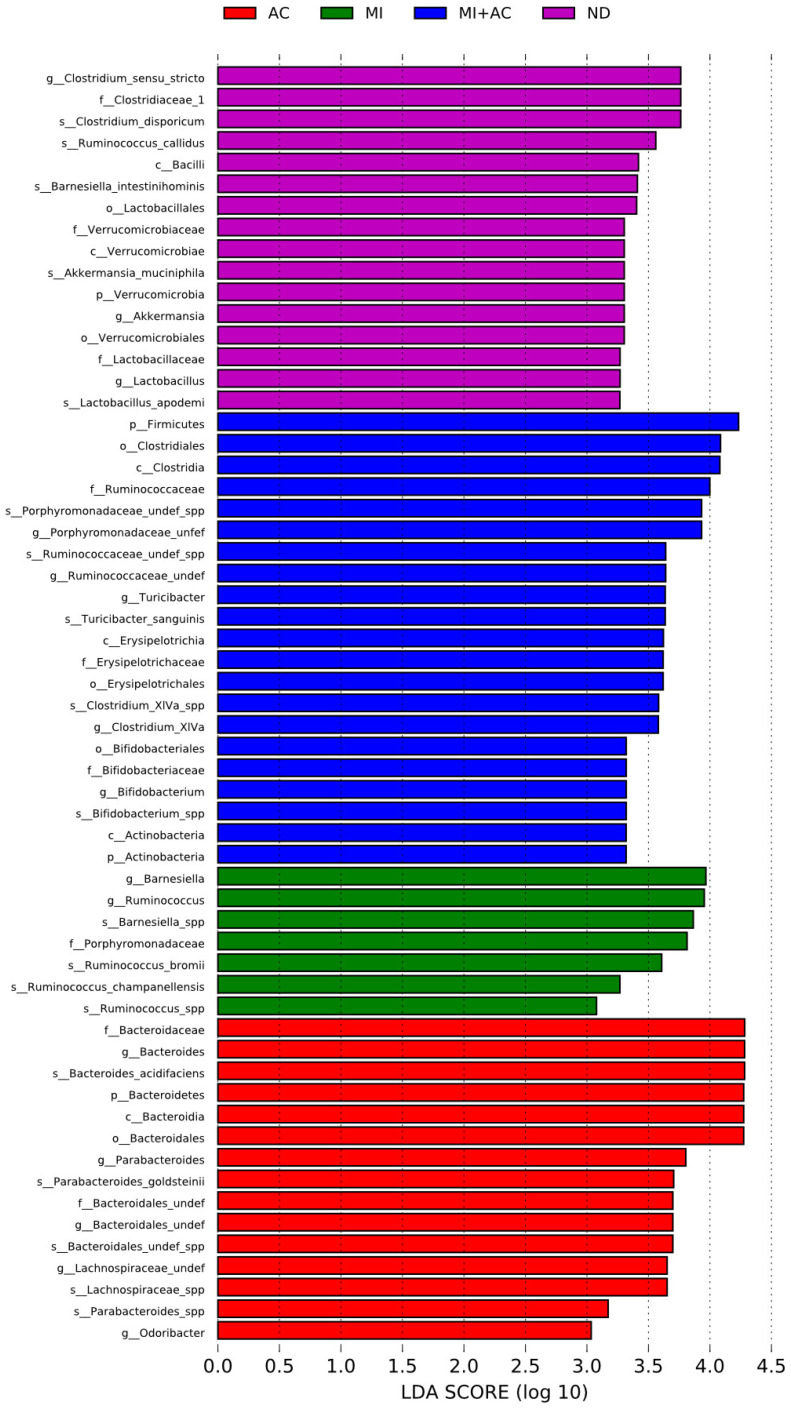
Effect of maternal minocycline administration (MI) and acetate supplementation (AC) on the offspring’s gut microbiome. Most enriched bacterial taxa in the ND (purple), MI (green), AC (red), and NI + AC (blue) groups are identified by the linear discriminant analysis effect size (LEfSe) analysis. The threshold on the linear discriminant (LDA) was set to 3. ND = control rats received vehicle; MI = rats received minocycline; AC = rats received acetate; MI + AC = rats received minocycline and acetate administration.

Results for the linear discriminant analysis effect size (LEfSe) algorithm to identify statistically significant biomarkers among groups are depicted in Figure 6. Minocycline administration caused a higher genus level of *Barnesiella* in the MI group. Acetate supplementation resulted in a higher proportion of genus *Odoribacter* in the AC group. Additionally, LEfSe analysis identified a higher abundance of genera *Bifidobacterium* and *Turicibacter* in the MI + AC group.

### 2.5. NO-Related Parameters

Considering nitric oxide (NO) deficiency is connected with hypertension of developmental origins [24], we further analyzed circulating NO-related parameters (Table 3). These parameters include l-citrulline (the precursor of l-arginine), l-arginine (the substrate for nitric oxide synthase), and asymmetric and symmetric dimethylarginine (ADMA and SDMA, both are NO synthase inhibitors). Minocycline administration displayed a negligible influence on plasma NO-related parameters. However, acetate supplementation caused decreases in plasma l-arginine and the ratio of l-arginine-to-ADMA, while an increase in ADMA in the AC group. Additionally, the MI + AC group had a higher l-citrulline and ADMA levels, but a lower ratio of l-arginine-to-ADMA compared to the MI group.

## 3. Discussion

Our study offers novel insights into the mechanisms underlying maternal minocycline-induced offspring hypertension and how acetate supplementation protects it, with special emphasis on gut microbiota and the kidneys. The significant findings can be summarized as follows: (1) Adult offspring born to dams exposed to minocycline during gestation and lactation developed hypertension at 12 weeks of age; (2) maternal acetate supplementation protected offspring against minocycline-induced hypertension; (3) maternal minocycline administration reduced plasma acetic acid level, which maternal acetate supplementation prevented; (4) acetate supplementation increased protein level of SCFA receptor GPR41 in the offspring kidneys; (5) combined minocycline and acetate administration significantly caused a reduction in inflammatory cytokine levels in offspring kidneys and gut microbiota shifts; (6) acetate supplementation protected minocycline-induced hypertension coinciding with the increase in genera *Roseburia*, *Bifidobacterium*, and *Coprococcus*.

Our results reconfirm previous findings revealing that early life antibiotics exposure can affect adult offspring’s BP, despite different antibiotics in various hypertensive animal models giving disparate results [17,25]. Minocycline is currently under investigation as a potential therapy for resistant hypertension [26]. Our current data showed that dams exposed to minocycline during gestation and lactation not only raised BP but also impaired renal function in their offspring. In terms of the translatability of these findings, the usefulness of minocycline for hypertension remains questionable at the present time. Future work determining whether maternal minocycline administration provides harmful or beneficial long-term effects in adult offspring is urgently warranted.

Consistent with prior research showing acetate treatment can avert the development of hypertension [21,22,23], this is the first report of maternal acetate supplementation preventing minocycline-induced hypertension and kidney injury in adult male offspring. Acetate supplementation prevented the reduction in circulating acetate induced by maternal minocycline administration, as well as upregulated GPR41 expression in offspring kidneys. Acetate is a ligand for GPR41, which has an anti-hypertensive effect [27]. According to our data, acetate-producing bacteria, such as *Roseburia* spp. and *Bifidobacterium* spp., were augmented by acetate supplementation. Hence, the beneficial effects of acetate supplementation against minocycline-induced hypertension are associated with its regulation of acetate-producing microbes, acetate production, and SCFA receptor expression.

Acetate and minocycline administration reduced BP in adults and might be related to the reduction in renal inflammation. Gut microbial dysbiosis is linked to aberrant immune responses accompanied by the abnormal production of inflammatory cytokines. Pro-inflammatory cytokines cause persistent low-grade inflammation, which takes part in the developmental programming of hypertension [3,14]. Previous research has shown that gut microbiota dysbiosis could promote T helper 17 (TH17) cell activation and stimulate the production of IL-6, IFN-γ, and IL-17A, resulting in hypertension [28]. Certain bacteria, such as *Bifidobacterium* spp., are able to diminish TH17 responses [29]. Here, we demonstrate that acetate supplementation augmented the genus abundance of *Bifidobacterium* and inhibits several pro-inflammatory cytokines production concurrently. In line with this view, our data revealed that acetate supplementation decreased IL-1B, IL-6, IFN-γ, and IL-17A concentrations in offspring kidneys, indicating a reduction in TH17-induced renal inflammation.

The advantageous effects of acetate are also relevant to gut microbial shifts. Studies involving hypertensive patients revealed that *Roseburia*, *Bifidobacterium*, and *Coprococcus* were present at lower levels while *Barnesiella* showed a higher abundance in the hypertension group compared to the control group [30]. According to our data, acetate supplementation increased genera *Roseburia*, *Bifidobacterium*, and *Coprococcus*, while decreasing *Barnesiella*.

Gut *Roseburia* spp. and *Bifidobacterium* spp. belong to commensal bacteria with probiotic properties [31,32]. In support of previous research indicating that gut microbiota-targeted therapies are able to treat or prevent the development of hypertension [19,20], we found acetate supplementation augmented genera *Roseburia* and *Bifidobacterium*, and reduced BP concurrently. Prior work reported a reduction in the level of *Coprococcus* spp. increases the risk of developing preeclampsia in pregnant women [33]. Thereby, further studies are needed to clarify whether acetate augmented *Coprococcus* abundance may have potential benefits for the prevention of hypertension.

Previous studies have demonstrated a positive correlation between the lactate-producing taxa and BP [34]. Similarly, the relative proportions of the lactate-producing bacteria were in higher abundance in the spontaneously hypertensive rats [6]. Our results showed that acetate supplementation prevented hypertension accompanied by a reduction in the abundance of *Barnesiella*, a lactate-producing bacteria. While a potential link between shifts in SCFA- and lactate-producing bacteria and BP regulation is not fully understood, our results provide important information on how acetate supplementation restores the balance of acetate- and SCFA-producing bacteria to avert the developmental programming of hypertension. Together, further studies are required to determine whether acetate protecting offspring against hypertension is directly relevant to alterations of these specific microbes.

Moreover, NO deficiency is another important mechanism reported to be behind hypertension of developmental origins [24]. Our current study did not detect the differences in NO-related parameters between the ND and MI groups, in spite of acetate supplementation decreasing the ratio of l-arginine-to-ADMA, an index of NO bioavailability. Our data agree with previous research revealing that minocycline inhibits NO production [35]. Considering that NO promotes vasodilatation, it is possible that the protective effect of acetate is not related to the NO pathway in this model.

Some limitations should be acknowledged in the current study. Firstly, we only focused on the kidneys. The impact of maternal acetate supplementation on other BP-controlled organs behind minocycline-induced hypertension is still little known. Secondly, we did not examine other gut microbiota-derived metabolites. Despite our study providing evidence for SCFAs in the developmental programming of hypertension, the role of other microbial metabolites remains largely unknown. So far, there is a lack of information regarding the simultaneous determination of all gut microbiota metabolites. There is a compelling need to improve the method for monitoring most microbial metabolites at one time and how these metabolites participate in the development of hypertension. Although the aberrant activation of the renin-angiotensin system (RAS) is also involved in the pathogenesis of minocycline-induced programmed hypertension [18], whether the beneficial effects of acetate are attributed to blocking the RAS or other mechanisms is worth further study. Lastly, we did not collect blood and feces from mother rats. Comparing mothers to offspring in a paired fashion might provide more details on how the gut microbiota and its metabolites of the mother rat could influence the progeny.

## 4. Materials and Methods

### 4.1. Animal Experimental Research Design

All experimental procedures were approved by the Institutional Animal Ethics Committee of Chang Gung Memorial Hospital, Kaohsiung, Taiwan (2019011001; approval date: 31 January 2019) in accordance with the guidelines for the Care and Use of Laboratory Animals of the National Institutes of Health. Sprague Dawley rats aged 8 weeks (BioLASCO Taiwan Co. Ltd., Taipei, Taiwan) were housed at 18–22 °C (12h light/dark cycle) and maintained ad libitum on water and standard chow in an AAALAC International-accredited animal facility. Pregnancies were achieved by putting one male and one female together in a breeding cage in the evening. Mating was confirmed if the vaginal plug was observed.

Pregnant rats were divided into four groups and treated as follows during pregnancy and lactation periods: (1) ND, vehicle; (2) MI, minocycline; (3) AC, acetate, and (4) MI + AC, minocycline plus acetate administration. Minocycline (50 mg/kg/day) was delivered via daily syringe feeding. Acetate supplementation was administered with magnesium acetate (200 mmol/L) in drinking water. The doses of minocycline and magnesium acetate used here were in reference to past rodent research, which was able to influence BP [18,23]. After birth, the litters were downsized to eight pups per mother to standardize maternal care. Subsequent experiments were performed with male-only offspring (N = 8/group), as males have more hypertension than females [36].

BP was measured using the CODA tail-cuff system (Kent Scientific Corporation, Torrington, CT, USA) monthly. Animals were allowed one week to habituate to the tail-cuff procedure. As stated before, BP measurements were accessed by the same research assistant on a blinded basis [18]. All rats were sacrificed until they were 12 weeks old. Fresh feces samples were frozen at −20 °C on collection and maintained at −80 °C freezer. Blood samples were collected in collection tubes containing heparin. Kidneys were harvested after perfusion, divided into medulla and cortex, and stored at −80 °C until analysis. The plasma creatinine level was determined by high-performance liquid chromatography (HPLC) [18].

### 4.2. Gas Chromatography-Mass Spectrometry (GC-MS)

We implemented gas chromatography–mass spectrometry (Agilent Technologies 7890B, Wilmington, DE, USA) equipped with an automated sampler to measure plasma levels SCFAs, including acetic acid, propionic acid, isobutyric acid, butyric acid, isovaleric acid, and valeric acid. We used 2-ethylbutiric acid as an internal standard. Chromatographic separation was performed using a DB-FFAP column (30 cm × 0.25 mm, 0.25 µm; Agilent Technologies), as we described previously [37]. Injections were held at 240 °C using a split ratio of 5:1 and an injection volume of 1 µL.

### 4.3. Western Blot

Renal cortical protein extracts were prepared for Western blotting with antibody incubation [18]. We used 10–15% polyacrylamide gels and separated them by electrophoresis. The separated proteins are then transferred onto a nitrocellulose membrane (GE Healthcare Bio-Sciences Corp., Piscataway, NJ, USA). After transfer, we stained the membrane with Ponceau S red (PonS) stain solution of 0.2% (*w*/*v*) in 1% acetic acid) to correct for variations in the total protein loading. Then, the membranes were imaged and saved in the TIFF file for later quantification. Following washing, we proceeded with the blocking. The membranes were blocked with 5% milk, and 0.05% Tween-20 in TBS followed by incubation with a primary antibody, washing, and incubation with a secondary antibody. A list of antibodies used for Western blotting is illustrated in Table 4. The bands were visualized using enhanced chemiluminescence reagent (PerkinElmer, Waltham, MA, USA) and quantified by densitometry (Quantity One Analysis software, Bio-Rad, Hercules, CA, USA). The protein level was presented as the integrated optical density (IOD)/PonS.

### 4.4. Determination of Cytokines in the Kidneys

Cytokines IL-1A, IL-1B, IL-2, IL-6, IL-17A, and IFN-γ in the renal cortical protein extracts were measured using a LEGENDplex^TM^ multiplex cytokine panel kit (BioLegend, SanDiego, CA, USA) [38]. Diluted samples were measured in duplicate using a BD FACS Canto II flow cytometer (BD Biosciences, San Jose, CA, USA). The fluorescent intensity of target cytokine concentration was identified in each bead set by its fluorescent color code. The data were quantified by LEGENDplex analysis software (BioLegend, San Diego, CA, USA).

### 4.5. Nitric Oxide-Related Parameters

We used the HP Agilent 1100 HPLC System (Agilent Technologies Inc., Santa Clara, CA, USA) with fluorescence detection using derivatization reaction utilizing O-phthalaldehyde/3-mercaptopropionic acid (OPA/3-MPA) to determine circulating NO-related parameters [37]. Homoarginine (Sigma-Aldrich, St. Louis, MO, USA) was used as the internal standard.

### 4.6. Gut Microbiota Composition

Bacterial DNA was extracted from stool samples and analyzed with meta-genomics using the methods stated before [18]. Amplicons were prepared based on the 16S Metagenomics Sequencing Library Preparation protocol (Illumina, San Diego, CA, USA). Sequencing and the processing of extracted data were performed at the Genomic and Proteomic Core Laboratory, Kaohsiung Chang Gung Memorial Hospital, where Illumina MiSeq platforms were applied to sequence the samples. Next-generation sequencing data were analyzed with the Microbial Genomics Module of CLC Genomics Workbench 9.5.4 (Qiagen, Stockach, Germany). Raw sequence data were processed in QIIME version 1.9.1 to obtain high-quality, clean tags. Chimera sequences were removed using the UCHIME algorithm. Using the UCLUST algorithm, sequence with ≥97% similarity was clustered into the same operational taxonomic units (OTUs). Based on a representative sequence alignment with Fast-Tree, the phylogenetic relationships were constructed. Alpha diversity was measured by the Shannon index. The β-diversity was accessed using the PCoA. We applied the LEfSe analysis to identify differentially enriched OTUs. A linear discriminant analysis (LDA) score threshold of >3 and *p* < 0.05 indicated significantly enriched microbial communities.

### 4.7. Statistical Analysis

All values are reported as mean ± the standard error of the mean (SEM). The calculations were analyzed with the Statistical Package for the Social Sciences software (SPSS Inc., Chicago, IL, USA). Comparisons within four groups were first analyzed by one-way analysis of variance (ANOVA), followed by Tukey’s post hoc test. Systolic BP was analyzed by a two-way repeated-measures analysis of variance and Tukey’s post hoc test. A *p*-value of less than 0.05 was regarded as statistically significant.

## 5. Conclusions

In sum, maternal acetate supplementation reshapes gut microbiota composition and regulates acetate and its receptor to provide protection against maternal minocycline-induced hypertension and kidney injury. Further in-depth research must be completed to greater understand the connection mechanisms between minocycline and acetate behind hypertension of developmental origins. Moving forward, our results are of significance to the development of interventions targeting early life programming in the prevention of hypertension.

## Figures and Tables

**Table 1:** Weight and blood pressure in 12-week-old male offspring.

Groups	ND	MI	AC	MI + AC
Mortality	0%	0%	12.5%	0%
Body weight (BW) (g)	367 ± 12 ^a^	335 ± 6 ^a^	205 ± 7 ^b^	242 ± 9 ^b^
Left kidney weight (g)	1.71 ± 0.06 ^a^	1.64 ± 0.03 ^a^	0.94 ± 0.04 ^c^	1.36 ± 0.08 ^b^
Left kidney weight/100 g BW	0.47 ± 0.01 ^b^	0.49 ± 0.01 ^b^	0.46 ± 0.01 ^b^	0.56 ± 0.02 ^a^
Systolic blood pressure (mmHg)	133.1 ± 0.4 ^b^	141.3 ± 2.2 ^a^	131.1 ± 0.9 ^b^	131.9 ± 0.8 ^b^
Diastolic blood pressure (mmHg)	90.3 ± 2.2 ^a^	96.3 ± 3.1 ^a^	84.6 ± 1.2 ^b^	88.4 ± 1.1 ^b^
Mean arterial pressure (mmHg)	104.2 ± 0.9 ^b^	111.4 ± 3.3 ^a^	100.4 ± 0.8 ^b^	103.2 ± 0.8 ^b^
Creatinine (μM)	14.94 ± 1.36 ^b^	18.63 ± 0.53 ^a^	17.07 ± 1.12 ^a^	16.46 ± 0.7 ^b^

Data are the mean ± SEM; *n* = 8/group; the letters a, b, and c denote significant differences between groups (*p* < 0.05, one-way ANOVA). ND = rats received vehicle (control); MI = rats received minocycline; AC = rats received acetate; MI + AC = rats received minocycline and acetate administration.

**Table 2:** Plasma SCFA levels in 12-week-old male offspring.

Groups	ND	MI	AC	MI + AC
Acetic acid (μM)	492.9 ± 8.11 ^b^	378.1 ± 12.39 ^c^	644.8 ± 27.23 ^a^	572.3 ± 20.93 ^a^
Propionic acid (μM)	8.44 ± 0.34	7.8 ± 0.37	8.63 ± 0.67	9.34 ± 0.6
Isobutyric acid (μM)	4.83 ± 0.15	3.94 ± 0.05	4.23 ± 0.05	3.99 ± 0.15
Butyric acid (μM)	5.6 ± 0.25	4.6 ± 0.56	4.09 ± 0.13	4.28 ± 0.22
Isovaleric acid (μM)	4.17 ± 0.17	3.4 ± 0.08	3.44 ± 0.05	3.33 ± 0.14
Valeric acid (μM)	4.82 ± 0.18	3.95 ± 0.12	4.39 ± 0.03	4.13 ± 0.06

Data are the mean ± SEM; *n* = 7–8/group; the letters a, b, and c denote significant differences between groups (*p* < 0.05, one-way ANOVA). ND = rats received vehicle (control); MI = rats received minocycline; AC = rats received acetate; MI + AC = rats received minocycline and acetate administration.

**Table 3:** Plasma levels of NO-related parameters in 12-week-old male offspring.

Groups	ND	MI	AC	MI + AC
l-Citrulline (μM)	98.4 ± 8.8 ^a^	81.2 ± 3.5 ^b^	94 ± 6.7 ^a^	110.3 ± 5.7 ^a^
l-Arginine (μM)	255.4 ± 7.2 ^a^	231.7 ± 7.8 ^a^	215.6 ± 10.5 ^b^	256.6 ± 10.5 ^a^
ADMA (μM)	1.7 ± 0.2 ^b^	1.5 ± 0.1 ^b^	2.1 ± 0.3 ^a^	2.2 ± 0.2 ^a^
SDMA (μM)	1.2 ± 0.1 ^a^	1 ± 0.1 ^b^	1.5 ± 0.3 ^a^	1.3 ± 0.1 ^a^
l-Arginine-to-ADMA ratio (μM/μM)	153.3 ± 9.6 ^a^	156.6 ± 9.7 ^a^	120.9 ± 7 ^b^	126.6 ± 11 ^b^

Data are the mean ± SEM; *n* = 7–8/group; ADMA = asymmetric dimethylarginine; SDMA = symmetric dimethylarginine. The letters a and b denote significant differences between groups (*p* < 0.05, one-way ANOVA). ND = rats received vehicle (control); MI = rats received minocycline; AC = rats received acetate; MI + AC = rats received minocycline and acetate administration.

**Table 4:** Antibodies used for Western blotting.

Antibody	Host	Source	Dilution
GPR41	Rabbit	USBiological, Swampscott, MA, USA	1:500
GPR43	Rabbit	Millipore, Burlington, MA, USA	1:500
GPR91	Rabbit	Novus Biologicals, Centennial, CO, USA	1:1000
Olfr78	Rabbit	Assay Biotech, San Francisco, CA, USA	1:500

## Data Availability

Data are contained within the article.
